# 1,5-Bis[1-(2,4-dihy­droxy­phen­yl)ethyl­idene]carbonohydrazide dimethyl­formamide disolvate

**DOI:** 10.1107/S1600536810043151

**Published:** 2010-10-30

**Authors:** Qing-Peng He, Bo Tan, Ze-Hua Lu

**Affiliations:** a College of Chemistry and Chemical Engineering, Liaocheng University, Shandong 252059, People’s Republic of China; b Liaocheng International Peace Hospital, Shandong 252059, People’s Republic of China

## Abstract

In the title compound, C_17_H_18_N_4_O_5_·2C_3_H_7_NO, two solvent mol­ecules are linked to the main mol­ecule *via* N—H⋯O and O—H⋯O hydrogen bonds, forming a hydrogen-bonded trimer. Intra­molecular O—H⋯N hydrogen bonds influence the mol­ecular conformation of the main mol­ecule, and the two benzene rings form a dihedral angle of 10.55 (18)°. In the crystal, inter­molecular O—H⋯O hydrogen bonds link hydrogen-bonded trimers into ribbons extending along the *b* axis.

## Related literature

For the biological activity of carbonohydrazide derivatives, see: Loncle *et al.* (2004 ▶); Li *et al.* (2004 ▶). For a related structure, see: Zukerman-Schpector *et al.* (2009 ▶).
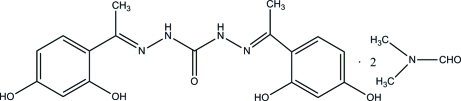


## Experimental

### 

#### Crystal data

C_17_H_18_N_4_O_5_·2C_3_H_7_NO
*M* *_r_* = 504.55
Monoclinic, 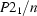

*a* = 11.3506 (11) Å
*b* = 9.0160 (7) Å
*c* = 24.953 (3) Å
β = 97.546 (1)°
*V* = 2531.5 (4) Å^3^
*Z* = 4
Mo *K*α radiation
μ = 0.10 mm^−1^
*T* = 298 K
0.50 × 0.37 × 0.35 mm

#### Data collection

Bruker SMART APEX CCD area-detector diffractometer
Absorption correction: multi-scan (*SADABS*; Sheldrick, 1996 ▶) *T*_min_ = 0.952, *T*_max_ = 0.966
12361 measured reflections
4466 independent reflections
2158 reflections with *I* > 2σ(*I*)
*R*_int_ = 0.055

#### Refinement

*R*[*F* ^2^ > 2σ(*F* ^2^)] = 0.051
*wR*(*F* ^2^) = 0.152
*S* = 1.03
4466 reflections
331 parameters
1 restraint
H-atom parameters constrained
Δρ_max_ = 0.30 e Å^−3^
Δρ_min_ = −0.27 e Å^−3^

### 

Data collection: *SMART* (Bruker, 2007 ▶); cell refinement: *SAINT* (Bruker, 2007 ▶); data reduction: *SAINT*; program(s) used to solve structure: *SHELXS97* (Sheldrick, 2008 ▶); program(s) used to refine structure: *SHELXL97* (Sheldrick, 2008 ▶); molecular graphics: *SHELXTL* (Sheldrick, 2008 ▶); software used to prepare material for publication: *SHELXTL*.

## Supplementary Material

Crystal structure: contains datablocks I, global. DOI: 10.1107/S1600536810043151/cv2779sup1.cif

Structure factors: contains datablocks I. DOI: 10.1107/S1600536810043151/cv2779Isup2.hkl

Additional supplementary materials:  crystallographic information; 3D view; checkCIF report

## Figures and Tables

**Table 1:** Hydrogen-bond geometry (Å, °)

*D*—H⋯*A*	*D*—H	H⋯*A*	*D*⋯*A*	*D*—H⋯*A*
O1—H1⋯N1	0.82	1.83	2.549 (3)	145
O3—H3⋯N4	0.82	1.84	2.562 (3)	146
O2—H2⋯O7	0.82	1.90	2.704 (4)	168
N2—H2′⋯O6	0.86	2.13	2.918 (3)	153
N3—H3′⋯O6	0.86	2.16	2.932 (3)	149
O4—H4⋯O5^i^	0.82	1.86	2.680 (3)	173

Symmetry code: (i) 
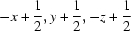
.

**Fig. 1.**
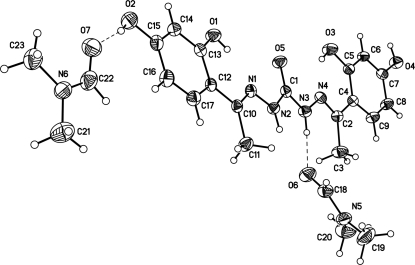
The content of asymmetric unit of the title compound showing the atomic numbering scheme and 30% probability displacement ellipsoids. Dashed lines denote intermolecular hydrogen bonds.

## supplementary crystallographic information

### Comment

Carbonohydrazide Schiff base derivatives are known to exhibit a wide range of interesting biological activities, including antibacterial antifungal, anticonvulsant, anticancer activities as well as herbicidal and fungicidal activity (Loncle *et al.*, 2004; Li *et al.*, 2004). Herewith we present the crystal structure of the title compound (I) - a new carbonohydrazide derivative.

In (I) (Fig. 1), the bond lengths and angles of the main molecule are normal and correspond to those observed in *N*'',*N*'''-bis (1-(2-hydroxyphenyl)ethylidene)carbonohydrazide dimethyl sulfoxide solvate (Zukerman-Schpector *et al.*, 2009). The intramolecular O—H···N hydrogen bonds (Table 1) influence the molecular conformation. Two DMF solvent molecules are linked to the main molecule *via* N—H···O and O—H···O hydrogen bonds (Table 1) forming a hydrogen-bonded trimer (Fig. 1). Intermolecular O—H···O hydrogen bonds (Table 1) link hydrogen-bonded trimers into ribbons extended along the *b* axis.

### Experimental

2, 4-Dihydroxylacetophenone (10.0 mmol) and carbohydrazide (5.0 mmol) were mixed in 50 ml flash. After 3h stirring at 373 K, the resulting mixture was cooled to room temperature, and recrystalized from DMF, and afforded the title compound as a crystalline solid.

### Refinement

All H atoms were placed in idealized positions (C—H 0.93–0.96 Å, N—H 0.86 Å, O—H 0.82 Å) and constrained to ride on their parent atoms, with *U*_iso_(H) = 1.2–1.5 *U*_eq_(parent atom).

### Crystal data

**Table:**

C_17_H_18_N_4_O_5_·2C_3_H_7_NO	*F*(000) = 1072
*M**_r_* = 504.55	*D*_x_ = 1.324 Mg m^−^^3^
Monoclinic, *P*2_1_/*n*	Mo *K*α radiation, λ = 0.71073 Å
*a* = 11.3506 (11) Å	Cell parameters from 2127 reflections
*b* = 9.0160 (7) Å	θ = 2.9–23.9°
*c* = 24.953 (3) Å	µ = 0.10 mm^−^^1^
β = 97.546 (1)°	*T* = 298 K
*V* = 2531.5 (4) Å^3^	Block, colourless
*Z* = 4	0.50 × 0.37 × 0.35 mm

### Data collection

**Table:**

Bruker SMART APEX CCD area-detector diffractometer	4466 independent reflections
Radiation source: fine-focus sealed tube	2158 reflections with *I* > 2σ(*I*)
graphite	*R*_int_ = 0.055
φ and ω scans	θ_max_ = 25.0°, θ_min_ = 2.8°
Absorption correction: multi-scan (*SADABS*; Sheldrick, 1996)	*h* = −11→13
*T*_min_ = 0.952, *T*_max_ = 0.966	*k* = −10→10
12361 measured reflections	*l* = −29→27

### Refinement

**Table:**

Refinement on *F*^2^	Primary atom site location: structure-invariant direct methods
Least-squares matrix: full	Secondary atom site location: difference Fourier map
*R*[*F*^2^ > 2σ(*F*^2^)] = 0.051	Hydrogen site location: inferred from neighbouring sites
*wR*(*F*^2^) = 0.152	H-atom parameters constrained
*S* = 1.03	*w* = 1/[σ^2^(*F*_o_^2^) + (0.0565*P*)^2^ + 0.4683*P*] where *P* = (*F*_o_^2^ + 2*F*_c_^2^)/3
4466 reflections	(Δ/σ)_max_ = 0.009
331 parameters	Δρ_max_ = 0.30 e Å^−^^3^
1 restraint	Δρ_min_ = −0.27 e Å^−^^3^

### Special details

**Table:**

Geometry. All e.s.d.'s (except the e.s.d. in the dihedral angle between two l.s. planes) are estimated using the full covariance matrix. The cell e.s.d.'s are taken into account individually in the estimation of e.s.d.'s in distances, angles and torsion angles; correlations between e.s.d.'s in cell parameters are only used when they are defined by crystal symmetry. An approximate (isotropic) treatment of cell e.s.d.'s is used for estimating e.s.d.'s involving l.s. planes.
Refinement. Refinement of *F*^2^ against ALL reflections. The weighted *R*-factor *wR* and goodness of fit *S* are based on *F*^2^, conventional *R*-factors *R* are based on *F*, with *F* set to zero for negative *F*^2^. The threshold expression of *F*^2^ > σ(*F*^2^) is used only for calculating *R*-factors(gt) *etc*. and is not relevant to the choice of reflections for refinement. *R*-factors based on *F*^2^ are statistically about twice as large as those based on *F*, and *R*- factors based on ALL data will be even larger.

### Fractional atomic coordinates and isotropic or equivalent isotropic displacement parameters (Å^2^)

**Table:**

	*x*	*y*	*z*	*U*_iso_*/*U*_eq_	
N3	0.5482 (2)	1.0132 (3)	0.38574 (9)	0.0413 (7)	
H3'	0.6233	1.0203	0.3961	0.050*	
N4	0.4960 (2)	1.0910 (3)	0.34129 (9)	0.0379 (7)	
N1	0.4829 (2)	0.7684 (3)	0.48746 (9)	0.0362 (7)	
N2	0.5404 (2)	0.8673 (3)	0.45843 (9)	0.0375 (7)	
H2'	0.6131	0.8921	0.4687	0.045*	
C1	0.4790 (3)	0.9250 (4)	0.41282 (12)	0.0352 (8)	
C2	0.5629 (3)	1.1733 (4)	0.31528 (12)	0.0371 (8)	
O1	0.29729 (18)	0.6107 (3)	0.49012 (9)	0.0542 (7)	
H1	0.3414	0.6680	0.4767	0.081*	
O4	0.34283 (19)	1.5034 (3)	0.13770 (9)	0.0537 (7)	
H4	0.2743	1.4758	0.1286	0.081*	
C4	0.5033 (3)	1.2567 (4)	0.26924 (11)	0.0346 (8)	
C12	0.4686 (3)	0.6037 (3)	0.55900 (11)	0.0348 (8)	
O5	0.37418 (19)	0.8984 (3)	0.39800 (9)	0.0519 (7)	
C10	0.5368 (3)	0.7104 (3)	0.53123 (12)	0.0353 (8)	
C7	0.3926 (3)	1.4211 (4)	0.18086 (12)	0.0405 (8)	
C6	0.3283 (3)	1.3208 (4)	0.20663 (12)	0.0406 (8)	
H14	0.2480	1.3068	0.1946	0.049*	
O3	0.31113 (19)	1.1458 (3)	0.27370 (9)	0.0607 (7)	
H3	0.3505	1.1027	0.2989	0.091*	
C14	0.2910 (3)	0.4568 (4)	0.56516 (13)	0.0465 (9)	
H16	0.2140	0.4307	0.5509	0.056*	
C13	0.3532 (3)	0.5593 (4)	0.53799 (12)	0.0391 (8)	
C9	0.5651 (3)	1.3599 (4)	0.24144 (13)	0.0476 (9)	
H18	0.6457	1.3738	0.2526	0.057*	
C5	0.3820 (3)	1.2408 (4)	0.25034 (12)	0.0378 (8)	
C17	0.5158 (3)	0.5373 (4)	0.60781 (13)	0.0472 (9)	
H20	0.5923	0.5634	0.6228	0.057*	
C15	0.3419 (3)	0.3934 (4)	0.61297 (14)	0.0460 (9)	
C16	0.4555 (3)	0.4360 (4)	0.63457 (13)	0.0500 (9)	
H22	0.4906	0.3958	0.6671	0.060*	
O2	0.2771 (2)	0.2926 (3)	0.63652 (10)	0.0647 (7)	
H2	0.3137	0.2667	0.6655	0.097*	
C8	0.5119 (3)	1.4409 (4)	0.19856 (13)	0.0482 (9)	
H24	0.5560	1.5090	0.1815	0.058*	
C11	0.6630 (3)	0.7461 (4)	0.55445 (13)	0.0512 (9)	
H26A	0.6754	0.8512	0.5530	0.077*	
H26B	0.7168	0.6964	0.5338	0.077*	
H26C	0.6771	0.7133	0.5913	0.077*	
C3	0.6951 (3)	1.1842 (4)	0.33107 (14)	0.0601 (11)	
H32A	0.7117	1.2473	0.3621	0.090*	
H32B	0.7310	1.2250	0.3016	0.090*	
H32C	0.7271	1.0872	0.3396	0.090*	
O6	0.7696 (2)	1.0156 (3)	0.46093 (10)	0.0652 (8)	
C18	0.8226 (3)	1.0859 (4)	0.49864 (15)	0.0531 (10)	
H18A	0.7916	1.0823	0.5313	0.064*	
N5	0.9189 (2)	1.1658 (3)	0.49747 (12)	0.0555 (8)	
C20	0.9664 (4)	1.1869 (5)	0.44770 (17)	0.0911 (15)	
H20A	0.9235	1.1262	0.4201	0.137*	
H20B	1.0488	1.1594	0.4523	0.137*	
H20C	0.9587	1.2893	0.4372	0.137*	
C19	0.9714 (4)	1.2474 (5)	0.54508 (17)	0.0898 (15)	
H19A	0.9311	1.2217	0.5753	0.135*	
H19B	0.9636	1.3519	0.5383	0.135*	
H19C	1.0540	1.2222	0.5530	0.135*	
N6	0.5058 (3)	0.0928 (4)	0.80215 (12)	0.0562 (8)	
C23	0.4210 (3)	−0.0012 (4)	0.82410 (14)	0.0664 (11)	
H23A	0.3454	0.0059	0.8019	0.100*	
H23B	0.4130	0.0302	0.8602	0.100*	
H23C	0.4482	−0.1020	0.8247	0.100*	
C22	0.4740 (4)	0.1708 (5)	0.75761 (18)	0.0661 (11)	
H22A	0.5326	0.2278	0.7448	0.079*	
C21	0.6253 (3)	0.0985 (5)	0.83068 (17)	0.0882 (14)	
H21A	0.6726	0.1650	0.8123	0.132*	
H21B	0.6598	0.0011	0.8318	0.132*	
H21C	0.6228	0.1332	0.8669	0.132*	
O7	0.3748 (2)	0.1750 (3)	0.73179 (11)	0.0717 (8)	

### Atomic displacement parameters (Å^2^)

**Table:**

	*U*^11^	*U*^22^	*U*^33^	*U*^12^	*U*^13^	*U*^23^
N3	0.0364 (15)	0.0483 (19)	0.0378 (15)	0.0007 (14)	−0.0007 (12)	0.0098 (14)
N4	0.0402 (15)	0.0406 (18)	0.0319 (15)	0.0063 (14)	0.0004 (12)	0.0065 (13)
N1	0.0425 (16)	0.0320 (17)	0.0338 (15)	0.0011 (13)	0.0043 (13)	0.0017 (13)
N2	0.0347 (14)	0.0403 (18)	0.0360 (15)	−0.0025 (13)	−0.0015 (12)	0.0051 (13)
C1	0.040 (2)	0.032 (2)	0.0328 (18)	0.0046 (16)	0.0008 (16)	−0.0022 (16)
C2	0.0377 (18)	0.042 (2)	0.0318 (18)	−0.0001 (17)	0.0061 (15)	−0.0038 (16)
O1	0.0406 (13)	0.0608 (19)	0.0587 (16)	−0.0030 (12)	−0.0026 (12)	0.0149 (13)
O4	0.0482 (14)	0.0626 (18)	0.0477 (14)	0.0000 (13)	−0.0038 (11)	0.0189 (13)
C4	0.0378 (18)	0.037 (2)	0.0282 (17)	0.0016 (16)	0.0029 (14)	−0.0016 (15)
C12	0.0416 (19)	0.030 (2)	0.0327 (18)	0.0023 (16)	0.0054 (15)	−0.0018 (15)
O5	0.0396 (13)	0.0582 (17)	0.0542 (14)	−0.0065 (12)	−0.0077 (11)	0.0107 (12)
C10	0.0405 (18)	0.030 (2)	0.0348 (18)	0.0011 (16)	0.0024 (15)	−0.0051 (16)
C7	0.0394 (19)	0.047 (2)	0.0347 (18)	0.0055 (17)	0.0017 (15)	0.0038 (17)
C6	0.0298 (17)	0.048 (2)	0.0434 (19)	−0.0005 (17)	0.0016 (15)	0.0085 (18)
O3	0.0432 (14)	0.072 (2)	0.0669 (18)	−0.0047 (14)	0.0067 (12)	0.0319 (14)
C14	0.042 (2)	0.042 (2)	0.057 (2)	−0.0006 (17)	0.0128 (17)	0.0010 (19)
C13	0.0417 (19)	0.036 (2)	0.0395 (19)	0.0052 (17)	0.0044 (16)	−0.0005 (16)
C9	0.0382 (19)	0.059 (3)	0.044 (2)	−0.0081 (18)	−0.0004 (16)	0.0053 (19)
C5	0.0373 (19)	0.041 (2)	0.0373 (18)	0.0001 (17)	0.0119 (15)	0.0050 (16)
C17	0.051 (2)	0.045 (2)	0.045 (2)	−0.0019 (18)	0.0012 (17)	−0.0013 (18)
C15	0.056 (2)	0.038 (2)	0.049 (2)	0.0041 (19)	0.0249 (19)	0.0009 (18)
C16	0.065 (2)	0.047 (2)	0.039 (2)	0.000 (2)	0.0053 (18)	0.0057 (18)
O2	0.0669 (17)	0.0618 (19)	0.0703 (19)	−0.0031 (15)	0.0271 (13)	0.0148 (15)
C8	0.042 (2)	0.054 (3)	0.047 (2)	−0.0096 (18)	0.0022 (16)	0.0161 (19)
C11	0.049 (2)	0.051 (2)	0.050 (2)	−0.0088 (18)	−0.0045 (16)	0.0054 (18)
C3	0.043 (2)	0.080 (3)	0.055 (2)	−0.005 (2)	−0.0022 (17)	0.019 (2)
O6	0.0573 (16)	0.076 (2)	0.0581 (16)	−0.0114 (15)	−0.0095 (13)	−0.0035 (15)
C18	0.047 (2)	0.061 (3)	0.051 (2)	−0.001 (2)	0.0053 (18)	0.008 (2)
N5	0.0405 (17)	0.061 (2)	0.065 (2)	−0.0069 (16)	0.0060 (15)	0.0056 (18)
C20	0.069 (3)	0.113 (4)	0.096 (3)	−0.006 (3)	0.025 (3)	0.036 (3)
C19	0.079 (3)	0.084 (4)	0.099 (3)	−0.032 (3)	−0.016 (3)	−0.016 (3)
N6	0.054 (2)	0.055 (2)	0.060 (2)	−0.0052 (17)	0.0098 (16)	−0.0024 (18)
C23	0.079 (3)	0.063 (3)	0.061 (2)	−0.005 (2)	0.025 (2)	0.005 (2)
C22	0.073 (3)	0.055 (3)	0.076 (3)	−0.007 (2)	0.033 (2)	0.000 (2)
C21	0.072 (3)	0.099 (4)	0.089 (3)	−0.017 (3)	−0.004 (2)	−0.012 (3)
O7	0.0652 (18)	0.078 (2)	0.0736 (19)	0.0075 (16)	0.0137 (15)	0.0123 (16)

### Geometric parameters (Å, °)

**Table:**

N3—C1	1.358 (4)	C15—O2	1.352 (4)
N3—N4	1.379 (3)	C15—C16	1.385 (4)
N3—H3'	0.8600	C16—H22	0.9300
N4—C2	1.295 (4)	O2—H2	0.8200
N1—C10	1.291 (3)	C8—H24	0.9300
N1—N2	1.368 (3)	C11—H26A	0.9600
N2—C1	1.358 (3)	C11—H26B	0.9600
N2—H2'	0.8600	C11—H26C	0.9600
C1—O5	1.223 (3)	C3—H32A	0.9600
C2—C4	1.462 (4)	C3—H32B	0.9600
C2—C3	1.504 (4)	C3—H32C	0.9600
O1—C13	1.359 (3)	O6—C18	1.225 (4)
O1—H1	0.8200	C18—N5	1.312 (4)
O4—C7	1.367 (3)	C18—H18A	0.9300
O4—H4	0.8200	N5—C20	1.430 (4)
C4—C9	1.402 (4)	N5—C19	1.456 (4)
C4—C5	1.403 (4)	C20—H20A	0.9600
C12—C17	1.400 (4)	C20—H20B	0.9600
C12—C13	1.403 (4)	C20—H20C	0.9600
C12—C10	1.465 (4)	C19—H19A	0.9600
C10—C11	1.507 (4)	C19—H19B	0.9600
C7—C6	1.374 (4)	C19—H19C	0.9600
C7—C8	1.380 (4)	N6—C22	1.325 (5)
C6—C5	1.381 (4)	N6—C23	1.443 (4)
C6—H14	0.9300	N6—C21	1.447 (4)
O3—C5	1.358 (3)	C23—H23A	0.9600
O3—H3	0.8200	C23—H23B	0.9600
C14—C15	1.380 (4)	C23—H23C	0.9600
C14—C13	1.392 (4)	C22—O7	1.222 (4)
C14—H16	0.9300	C22—H22A	0.9300
C9—C8	1.369 (4)	C21—H21A	0.9600
C9—H18	0.9300	C21—H21B	0.9600
C17—C16	1.367 (4)	C21—H21C	0.9600
C17—H20	0.9300		
			
C1—N3—N4	118.9 (2)	C15—O2—H2	109.5
C1—N3—H3'	120.6	C9—C8—C7	119.6 (3)
N4—N3—H3'	120.6	C9—C8—H24	120.2
C2—N4—N3	118.5 (3)	C7—C8—H24	120.2
C10—N1—N2	120.4 (2)	C10—C11—H26A	109.5
C1—N2—N1	117.7 (3)	C10—C11—H26B	109.5
C1—N2—H2'	121.1	H26A—C11—H26B	109.5
N1—N2—H2'	121.1	C10—C11—H26C	109.5
O5—C1—N3	124.6 (3)	H26A—C11—H26C	109.5
O5—C1—N2	123.4 (3)	H26B—C11—H26C	109.5
N3—C1—N2	112.0 (3)	C2—C3—H32A	109.5
N4—C2—C4	116.5 (3)	C2—C3—H32B	109.5
N4—C2—C3	122.5 (3)	H32A—C3—H32B	109.5
C4—C2—C3	121.0 (3)	C2—C3—H32C	109.5
C13—O1—H1	109.5	H32A—C3—H32C	109.5
C7—O4—H4	109.5	H32B—C3—H32C	109.5
C9—C4—C5	115.7 (3)	O6—C18—N5	126.4 (4)
C9—C4—C2	121.3 (3)	O6—C18—H18A	116.8
C5—C4—C2	123.0 (3)	N5—C18—H18A	116.8
C17—C12—C13	115.8 (3)	C18—N5—C20	120.1 (3)
C17—C12—C10	121.8 (3)	C18—N5—C19	120.8 (3)
C13—C12—C10	122.4 (3)	C20—N5—C19	118.7 (3)
N1—C10—C12	116.4 (3)	N5—C20—H20A	109.5
N1—C10—C11	124.1 (3)	N5—C20—H20B	109.5
C12—C10—C11	119.6 (3)	H20A—C20—H20B	109.5
C6—C7—O4	122.4 (3)	N5—C20—H20C	109.5
C6—C7—C8	119.7 (3)	H20A—C20—H20C	109.5
O4—C7—C8	117.9 (3)	H20B—C20—H20C	109.5
C7—C6—C5	120.4 (3)	N5—C19—H19A	109.5
C7—C6—H14	119.8	N5—C19—H19B	109.5
C5—C6—H14	119.8	H19A—C19—H19B	109.5
C5—O3—H3	109.5	N5—C19—H19C	109.5
C15—C14—C13	121.0 (3)	H19A—C19—H19C	109.5
C15—C14—H16	119.5	H19B—C19—H19C	109.5
C13—C14—H16	119.5	C22—N6—C23	120.5 (3)
O1—C13—C14	116.5 (3)	C22—N6—C21	121.9 (4)
O1—C13—C12	122.4 (3)	C23—N6—C21	117.6 (3)
C14—C13—C12	121.1 (3)	N6—C23—H23A	109.5
C8—C9—C4	122.9 (3)	N6—C23—H23B	109.5
C8—C9—H18	118.5	H23A—C23—H23B	109.5
C4—C9—H18	118.5	N6—C23—H23C	109.5
O3—C5—C6	116.3 (3)	H23A—C23—H23C	109.5
O3—C5—C4	122.1 (3)	H23B—C23—H23C	109.5
C6—C5—C4	121.6 (3)	O7—C22—N6	126.1 (4)
C16—C17—C12	123.5 (3)	O7—C22—H22A	116.9
C16—C17—H20	118.2	N6—C22—H22A	116.9
C12—C17—H20	118.2	N6—C21—H21A	109.5
O2—C15—C14	117.7 (3)	N6—C21—H21B	109.5
O2—C15—C16	123.3 (3)	H21A—C21—H21B	109.5
C14—C15—C16	119.0 (3)	N6—C21—H21C	109.5
C17—C16—C15	119.7 (3)	H21A—C21—H21C	109.5
C17—C16—H22	120.2	H21B—C21—H21C	109.5
C15—C16—H22	120.2		

### Hydrogen-bond geometry (Å, °)

**Table:**

*D*—H···*A*	*D*—H	H···*A*	*D*···*A*	*D*—H···*A*
O1—H1···N1	0.82	1.83	2.549 (3)	145
O3—H3···N4	0.82	1.84	2.562 (3)	146
O2—H2···O7	0.82	1.90	2.704 (4)	168
N2—H2'···O6	0.86	2.13	2.918 (3)	153
N3—H3'···O6	0.86	2.16	2.932 (3)	149
O4—H4···O5^i^	0.82	1.86	2.680 (3)	173

Symmetry codes: (i) −*x*+1/2, *y*+1/2, −*z*+1/2.
